# Correction to: Revolutionizing GPCR–ligand predictions: DeepGPCR with experimental validation for high-precision drug discovery

**DOI:** 10.1093/bib/bbae427

**Published:** 2024-08-15

This is a correction to: Haiping Zhang, Hongjie Fan, Jixia Wang, Tao Hou, Konda Mani Saravanan, Wei Xia, Hei Wun Kan, Junxin Li, John Z H Zhang, Xinmiao Liang, Yang Chen, Revolutionizing GPCR–ligand predictions: DeepGPCR with experimental validation for high-precision drug discovery, *Briefings in Bioinformatics*, Volume 25, Issue 4, July 2024, bbae281, https://doi.org/10.1093/bib/bbae281

In the originally published version of this manuscript, the information regarding first co-authorship of authors Haiping Zhang and Hongjie Fan was omitted in error.

This has been corrected.

