# Supplementary material for: Revolutionizing GPCR–ligand predictions: DeepGPCR with experimental validation for high-precision drug discovery
Source: Brief Bioinform. 2024 Jun 12;25(4):bbae281. doi: 10.1093/bib/bbae281 (PMC11167311; doi:10.1093/bib/bbae281)
Supplement: Supplementary_materials_n_bbae281 [file supplementary_materials_n_bbae281.docx]

**Supplementary Material Section 1**

**Schrödinger glide docking procedure**

We used Schrödinger glide docking to further screening compounds based on the predicted the binding affinity. First, the ligands underwent geometric optimization using the Ligprep module. The ligand energy was minimized using the OPLS 2005 force field, and all its ionized states were generated at pH 7.4. Subsequently, a single, low-energy three-dimensional structure of the ligand was generated, while preserving its original chiral state. Hydrogen atoms were added to the protein, and the system was optimized at pH 7.4 using the OPLS-u-2005 force field. The receptor grid was generated based on the geometric center coordinates of the protein’s ligands, with a size of 26 Å x 26 Å x 26 Å. The docking process adopted default settings such as standard precision, flexible sampling, and no constraints.

**Supplementary Material Section 2**

**Performance metrics for binary classification model**

To assess model performance, we employed various evaluation metrics including AUC (Area Under the ROC Curve), TPR (True Positive Rate), Precision, Accuracy, MCC (Matthews Correlation Coefficient), and F1 score. AUC, representing the area under the ROC (Receiver Operating Characteristic) curve, ranges from 0.5 to 1, where 0.5 denotes a random classifier, and 1 indicates a perfect classifier. TPR, known as recall, refers to the proportion of true positive instances that are correctly predicted as positive. Precision measures the ratio of true positives to the sum of true positives and false positives, representing the proportion of true positives to the total predicted positives by the model. Accuracy, ranging from 0 to 1, is the ratio of correctly classified samples out of the total number of samples. MCC, a correlation coefficient, quantifies the relationship between the actual and predicted binary classifications. It takes values between -1 to 1. A value of -1 indicates a perfect negative correlation, 1 reveals a perfect positive correlation, and 0 indicates no correlation. The F1 score, an essential metric for binary classification models, combines Precision, and Recall to provide a balanced perspective on the performance of model.

It should be noted that the aforementioned performance metrics are tailored to evaluate binary classification prediction ranging from 0 to1.In contrast, Schrödinger and Autodock Vina predicted value of linear; hence, we used -6 kcal/mol as the cutoff, those scores greater than -6 kcal/mol was assigned value 0, indicating non-bind, and those scores equal to or less than -6 kcal/mol were assigned a value of 1, representing binding capability. This approach enables the evaluation of performance with the above evaluation metrics.

**Performance metrics for the** **Regression model**

We used RMSE (Root Mean Squared Error), MSE (Mean Squared Error), Pearson (Pearson correlation coefficient), Spearman (Spearman correlation coefficient) and C-index (Concordance Index, CI) to evaluate the performance of the regression model. RMSE is computed as the square root of the average squared differences between the predicted and actual values. MSE is the average of the squared differences. The Pearson correlation measures the linear relationship between these predicted and actual values. Spearman correlation is a non-parametric assessment of the monotonic relationship between variables. C-index measures the ability of a model to rank the observed outcomes correctly in terms of their relative risk or event occurrence probabilities.

**Supplementary Material Section 3**

**The interactions of GPR35 with F545-1970 S948-0241 and Zaprinast, respectively.**

The interactions between GPR35 and F545-1970 are shown in **Figure 6B**. The control compounds and proteins predominantly engage in hydrophobic, electrostatic, and polar interactions. LEU258, TYR259, LEU80, and TYR96 are primarily involved in hydrophobic interactions with the cyclohexane portion of compound F545-1970. Notably, TYR96 establishes π-π interactions with the cyclohexane ring of the compound. ARG100 and the 1,2,4-oxydiazole moiety of compound F545-1970 engage in an electrostatic interaction. Additionally, the oxygen atom in the structure of SER165 and the phenyl group of PHE163 form hydrogen bonds with the nitrogen atom of compound F545-1970.

The interactions between GPR35 and S948-0241 are shown in **Figure 6C**. The primary interactions between the control compounds and proteins are hydrodynamic and electrostatic in nature. LEU258, TYR259, LEU13, and TYR96 predominantly engage in hydrophobic interactions with the cyclohexane moiety of compound S948-0241, with TYR96 specifically forming π-alkyl interactions with the methylene groups of the compound. Furthermore, ARG100 and ARG151 establish electrostatic interactions with the positively charged elements of the trifluoromethyl group in the compound, namely the fluorine atoms and the nitrogen atom of the cyanide group.

The interaction between GPR35 and known active compounds Zaprinast are shown in **Figure 6D**. The control compounds and proteins predominantly engage in hydrodynamic and electrostatic interactions. LEU258, TYR259, PHE163, and TRP96 primarily establish hydrophobic interactions with the carbon atoms in the backbone of the control compounds. Conversely, LYS263 and ARG100 are involved in electrostatic interactions with the framework of the control compounds. Additionally, LYS236 engages in a salt bridge interaction with the nitrogen atom located at position 2 of the triazole.

**Supplementary Material Section 4**

**Screening against three targets,** **O14626, O95800, and Q9HC97**

Overall, we selected three GPCR proteins, namely O14626 (GPR171), O95800 (GPR75), and Q9HC97 (GPR35), to showcase the applications of our DeepGPCR model in screening potential therapeutic compounds. These three GPCR proteins are all potential therapeutic targets for various diseases, including cancer, metabolic syndrome, and related disorders. For instance, GPR171 is a T-cell checkpoint that plays a crucial role in tumor immunity, inhibiting it can be a potential anticancer drug ^1^. Similarly, targeting the 20-HETE/GPR75 pathway has been discovered as a new, highly druggable potential target in the metabolic syndrome ^2^ and is also promising in interfering with prostate tumor cell malignant progression ^3^. Lastly, GPR35 is a potential target for various diseases ^4^, including cancer ^5^. Using our DeepGPCR model, we screened 102,472, 102,563, and 102,592 candidates with a score of ≥ 0.999 for O14626, O95800, and Q9HC97, respectively. We then grouped each protein's candidates into 1000 clusters and obtained potential representative compounds. Further clustering these 1000 compounds into 30 clusters helped us identify the final representative compounds depicted in Figure S6. The clustering analysis employed the default Ward's Hierarchical Agglomerative Clustering Method ^6,7^ implemented in Clusfps (https://github.com/kaiwang0112006/clusfps).

Considering the DeepGPCR model cannot directly provide GPCR-ligand binding conformation, we relied on the Schrödinger to dock the candidates into the protein targets. For O14626 and Q9HC97, we directly docked the 1000 representative candidates to their predicted pockets. However, the predicted binding cavity of O95800 was much narrower due to structural constraints according to our visual observation of the pocket region of its 3D structure. We tested the docking of molecules of different sizes. We observed that larger molecules had worse docking scores, which is consistent with our observation that the binding cavity is too narrow for large molecules to bind. Therefore, we selected 1923 compounds with a molecular weight ≤300 from its 102,563 candidate compounds for docking.

**Supplementary Material Section 5**

**Detailed procedure of pocket MD and metadynamics simulation**

The initial protein-compound complexes were from the top score conformation Schrödinger docking. The ligand was edited by PyMOL software ^8^ to make it in the correct protonation state at pH 7.

To make the simulation closer to GPCR environment, we have carried MD for GPR35-compound complexes embedded in lipid membrane. We preparation of the simulation system by building the GPR35-membrane-water-small molecule complex and solvate it using an appropriate solvent model. The system should be energy minimized and equilibrated using molecular dynamics simulation before the production run. Metadynamics simulations can estimate binding free energy calculation to explore whether protein-ligand will bind in solution. Metadynamics relies on adding a bias potential to sample the free energy landscape along a specific collective variable of interest ^9^,^10^. Note that the binding free energy calculations from Metadynamics may only be suitable for detecting the general trend of binding in virtual screening.

The MD simulation was carried out by Gromacs with AMBER-99 force field ^11,12^. The lipid PDB structure file (DPPC_293K.pdb)) and parameter files (DPPC.itp) are downloaded from Slipids websever (<http://www.fos.su.se/~sasha/SLipids/Downloads.html>) ^13–15^. The topology of the ligand and the partial charges of the ligand were generated by ACPYPE ^16^, which relies on Antechamber ^17^. The procedure to prepare the initial protein-ligand-lipid membrane with solvent and counter ion are largely follow the Gromac tutorial (http://www.mdtutorials.com/gmx/membrane_protein/02_topology.html), the major difference is that the forcefield we used was AMBER instead of GROMOS in the tutorial. We used TIP3P water molecules ^18^, and the counter ions were added to neutralize the total charge using the Gromacs program tool ^19^. The long-range electrostatic interactions under the periodic boundary conditions were calculated with the Particle Mesh Ewald approach ^20^. A cutoff of 10 Å was used for van der Waals non-bonded interactions. Covalent bonds involving hydrogen atoms were constrained by applying the LINCS algorithm ^21^.

We performed the energy minimization steps with a step-size of 0.001ns, 100 ps simulation with an isothermal-isovolumetric ensemble (NVT), and 10ns simulation with the isothermal-isobaric ensemble (NPT) for water equilibrium. After that, a 40ns NPT production run (step size 2 fs) was carried out. The Parrinello-Rahman barostat and the modified Berendsen thermostat were used for simulation with a fixed temperature of 308 K and a pressure of 1 atm. RMSD and hydrogen bond number of the trajectory were calculated using Gromacs tools.

The simulation continued using the metadynamics approach to explore the free energy landscape. We carried 40ns metadynamics simulation with Plumed^22^ patched Gromacs. The protein-ligand complex's interface coordination number of atoms was used as a collective variable (CV). The protein-ligand interface coordination numbers correlate with the numbers of atom contact, and a larger coordination number usually indicates that the protein-ligand is binding.

The coordination number C is defined as follows by Plumed:

$C=\sum_{i\in A}\sum_{j\in B}S_{ij}$ (1) and

$S_{ij}=\frac{1-\left(\frac{r_{ij}-d_{0}}{r_{0}}\right)^{n}}{1-\left(\frac{r_{ij}-d_{0}}{r_{0}}\right)^{m}}$ (2)

In the simulation, n was 8, m was 12, $d_{0}$ was 0 nm, and $r_{0}$ was 0.25 nm. $d_{0}$ is a parameter of the switching function. $r_{ij}$ is the distance between atom i and atom j. The degrees of contact between two groups of atoms can be estimated by the above function(1) ^22^. Metadynamics simulation for each protein-ligand system was performed for 40 ns. During the metadynamics simulation, Gaussian values were deposited every 1 ps with a height of 0.3 kJ/mol. The widths of the Gaussians were 5 for the coordination number. The free energy landscapes of the metadynamics simulations along the CV were generated by the Plumed program and plotted using Gnuplot ^23^.

**Reference**

1. Fujiwara, Y. *et al.* The GPR171 pathway suppresses T cell activation and limits antitumor immunity. *Nat. Commun.* (2021) doi:10.1038/s41467-021-26135-9.

2. Murtaza, B., Asghar, F. & Patoli, D. GPR75: An exciting new target in metabolic syndrome and related disorders. *Biochimie* at https://doi.org/10.1016/j.biochi.2022.01.005 (2022).

3. Cárdenas, S. *et al.* GPR75 receptor mediates 20-HETE-signaling and metastatic features of androgen-insensitive prostate cancer cells. *Biochim. Biophys. Acta - Mol. Cell Biol. Lipids* (2020) doi:10.1016/j.bbalip.2019.158573.

4. Quon, T., Lin, L. C., Ganguly, A., Tobin, A. B. & Milligan, G. Therapeutic Opportunities and Challenges in Targeting the Orphan G Protein-Coupled Receptor GPR35. *ACS Pharmacology and Translational Science* at https://doi.org/10.1021/acsptsci.0c00079 (2020).

5. Pagano, E. *et al.* Activation of the GPR35 pathway drives angiogenesis in the tumour microenvironment. *Gut* (2022) doi:10.1136/gutjnl-2020-323363.

6. Murtagh, F. & Legendre, P. Ward’s Hierarchical Agglomerative Clustering Method: Which Algorithms Implement Ward’s Criterion? *J. Classif.* **31**, 274–295 (2014).

7. Murtagh, F. & Contreras, P. Algorithms for hierarchical clustering: An overview. *Wiley Interdiscip. Rev. Data Min. Knowl. Discov.* (2012) doi:10.1002/widm.53.

8. DeLano, W. L. Pymol: An open-source molecular graphics tool. *CCP4 Newsl. Protein Crystallogr.* (2002).

9. Laio, A. & Gervasio, F. L. Metadynamics: a method to simulate rare events and reconstruct the free energy in biophysics, chemistry and material science. *Reports Prog. Phys.* **71**, 126601 (2008).

10. Saleh, N., Ibrahim, P., Saladino, G., Gervasio, F. L. & Clark, T. An Efficient Metadynamics-Based Protocol To Model the Binding Affinity and the Transition State Ensemble of G-Protein-Coupled Receptor Ligands. *J. Chem. Inf. Model.* **57**, 1210–1217 (2017).

11. Hess, B., Kutzner, C. & Spoel, D. Van Der. GROMACS 4: algorithms for highly efficient, load-balanced, and scalable molecular simulation. *J. Chem.* (2008).

12. Hornak, V. & Simmerling, C. Generation of accurate protein loop conformations through low-barrier molecular dynamics. *Proteins Struct. Funct. Genet.* (2003) doi:10.1002/prot.10363.

13. Jämbeck, J. P. M. & Lyubartsev, A. P. Derivation and Systematic Validation of a Refined All-Atom Force Field for Phosphatidylcholine Lipids. *J. Phys. Chem. B* **116**, 3164–3179 (2012).

14. Jämbeck, J. P. M. & Lyubartsev, A. P. An Extension and Further Validation of an All-Atomistic Force Field for Biological Membranes. *J. Chem. Theory Comput.* **8**, 2938–2948 (2012).

15. Grote, F. & Lyubartsev, A. P. Optimization of Slipids Force Field Parameters Describing Headgroups of Phospholipids. *J. Phys. Chem. B* **124**, 8784–8793 (2020).

16. Sousa Da Silva, A. W. & Vranken, W. F. ACPYPE - AnteChamber PYthon Parser interfacE. *BMC Res. Notes* (2012) doi:10.1186/1756-0500-5-367.

17. Wang, J., Wang, W., Kollman, P. A. & Case, D. A. Automatic atom type and bond type perception in molecular mechanical calculations. *J. Mol. Graph. Model.* **25**, 247–260 (2006).

18. Jorgensen, W. L., Chandrasekhar, J., Madura, J. D., Impey, R. W. & Klein, M. L. Comparison of simple potential functions for simulating liquid water. *J. Chem. Phys.* **79**, 926–935 (1983).

19. Van Der Spoel, D. *et al.* GROMACS: Fast, flexible, and free. *Journal of Computational Chemistry* at https://doi.org/10.1002/jcc.20291 (2005).

20. Darden, T., York, D. & Pedersen, L. Particle mesh Ewald: An N ⋅log( N ) method for Ewald sums in large systems. *J. Chem. Phys.* **98**, 10089–10092 (1993).

21. Hess, B., Bekker, H., Berendsen, H. J. C. & Fraaije, J. G. E. M. LINCS: A linear constraint solver for molecular simulations. *J. Comput. Chem.* **18**, 1463–1472 (1997).

22. Tribello, G. A., Bonomi, M., Branduardi, D., Camilloni, C. & Bussi, G. PLUMED 2: New feathers for an old bird. *Comput. Phys. Commun.* (2014) doi:10.1016/j.cpc.2013.09.018.

23. Williams, T. *et al.* Gnuplot 4.6. *Softw. Man.* (2012).

**Supplementary Figures:**


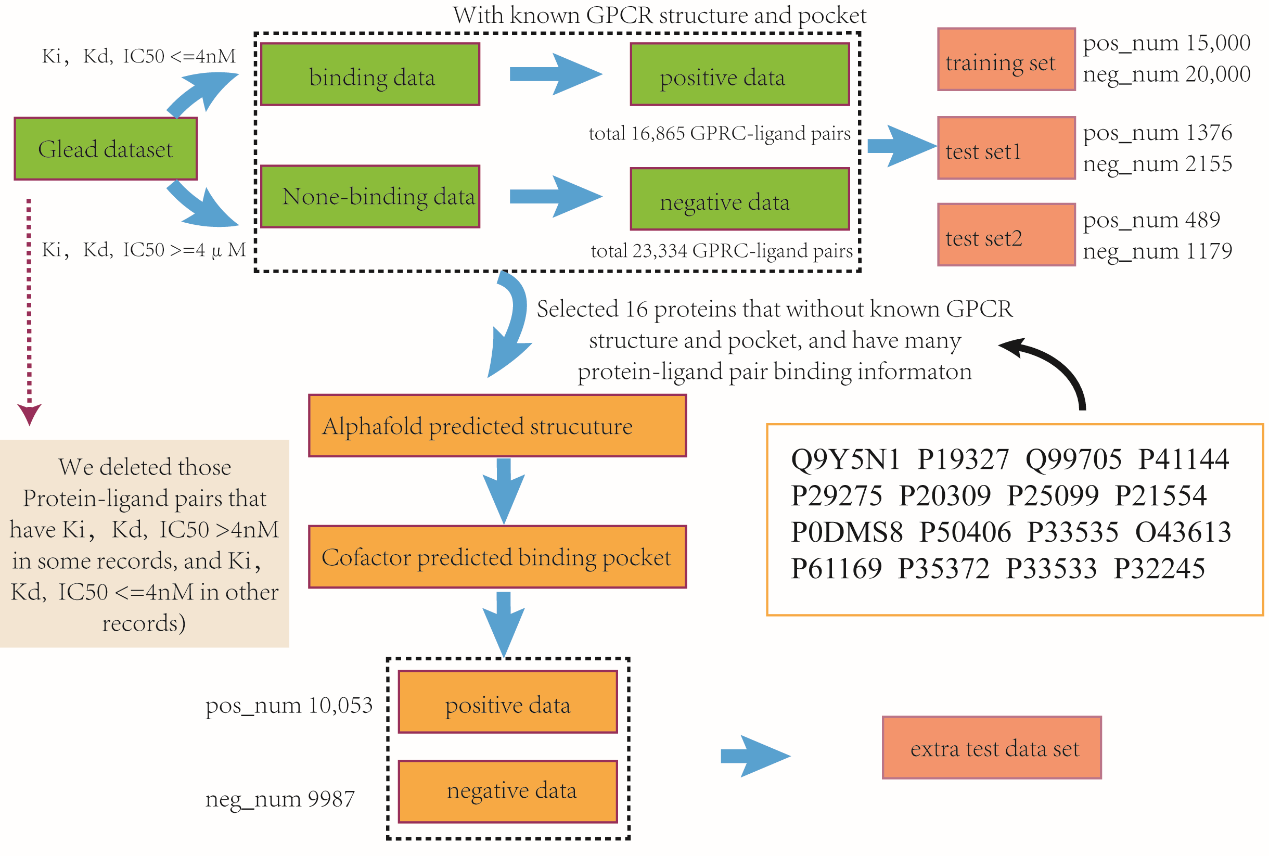


**Figure S1. Data preparation for training and test.**


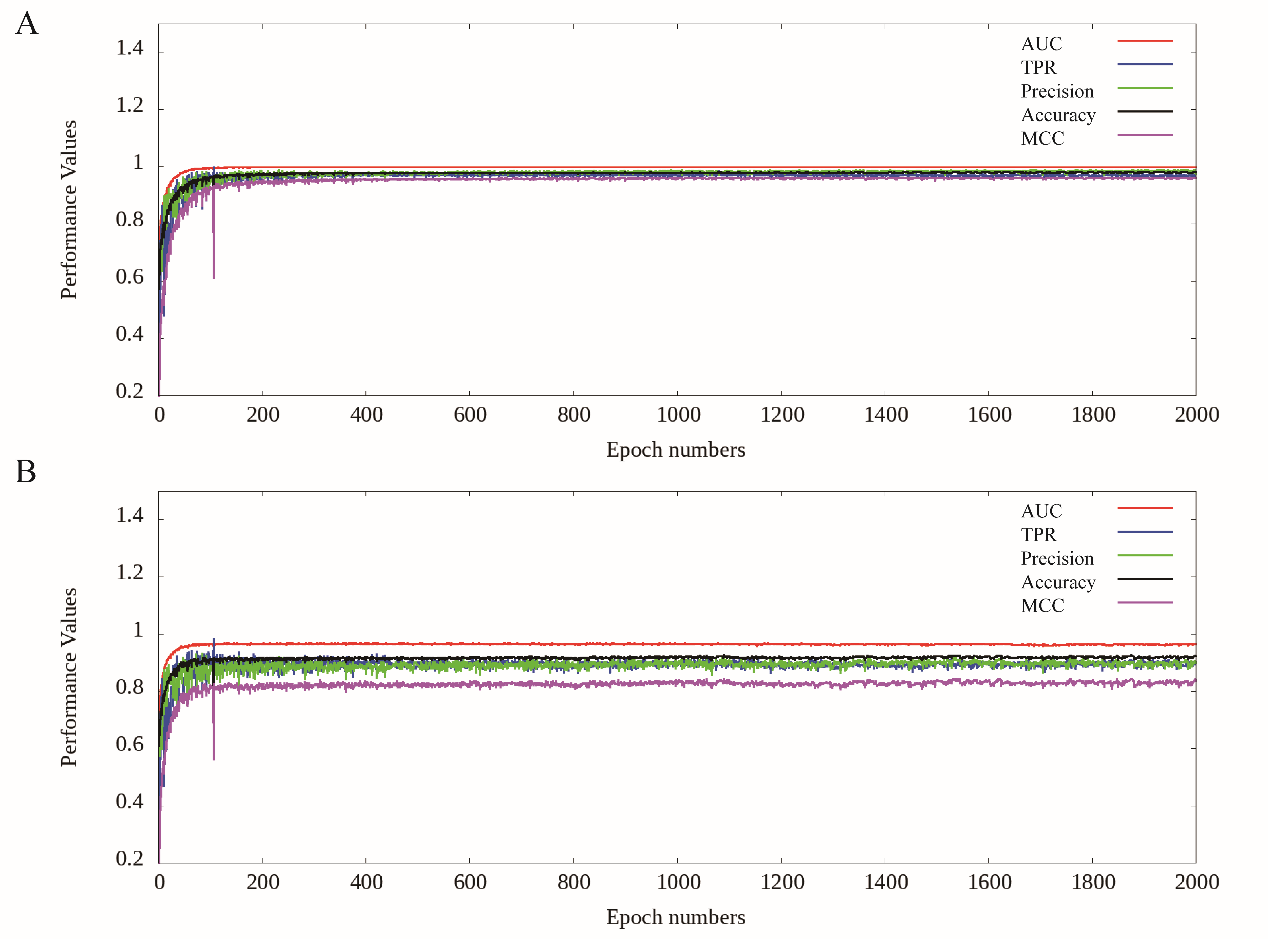


**Figure S2**. **The plot of model performance on the training and testing set along different training epochs.**

**
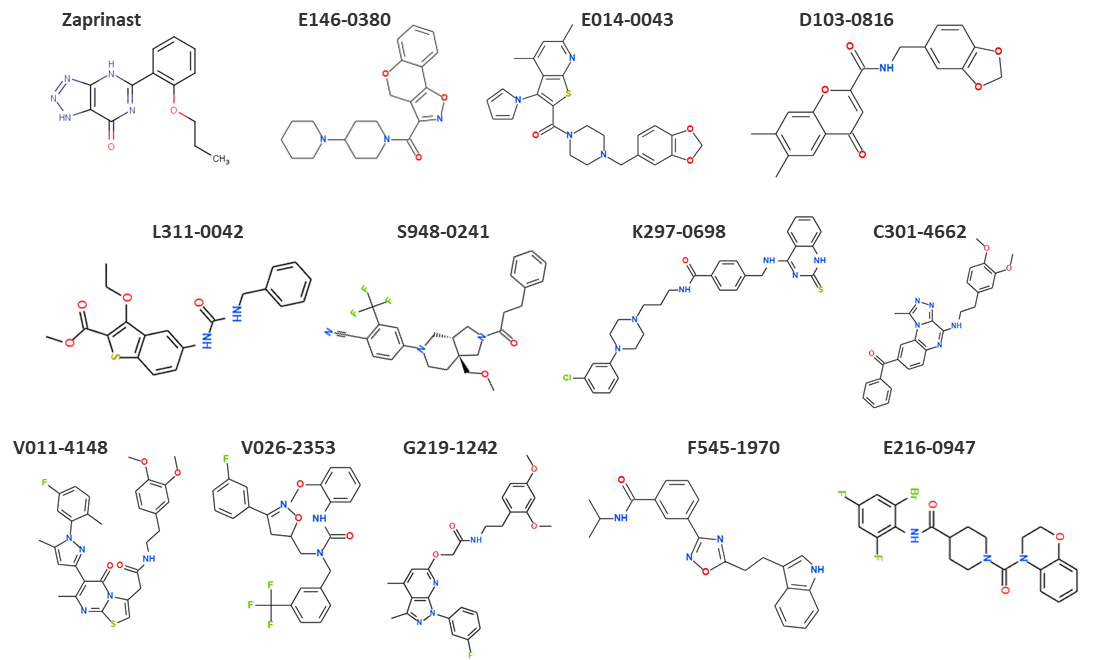
**

**Figure S3. Chemical structures of 12 selective candidates for GPR35.**


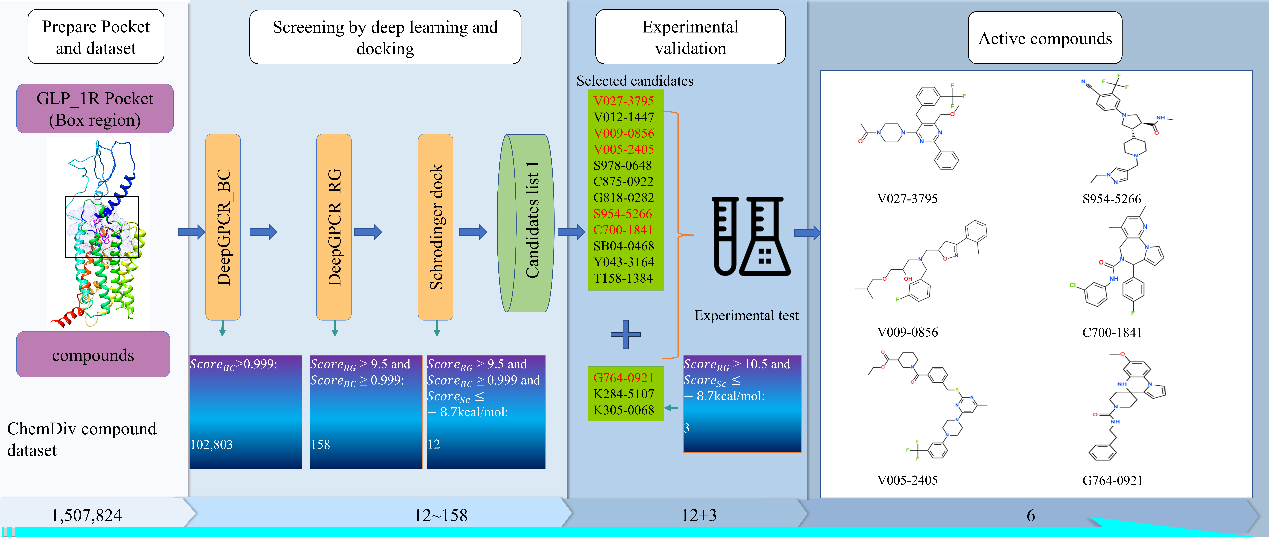


**Figure S4. GLP_1R screening pipeline and Identification of Active Compounds.** Schematic representation of the stepwise screening process leading to the discovery of 6 active molecules.

**
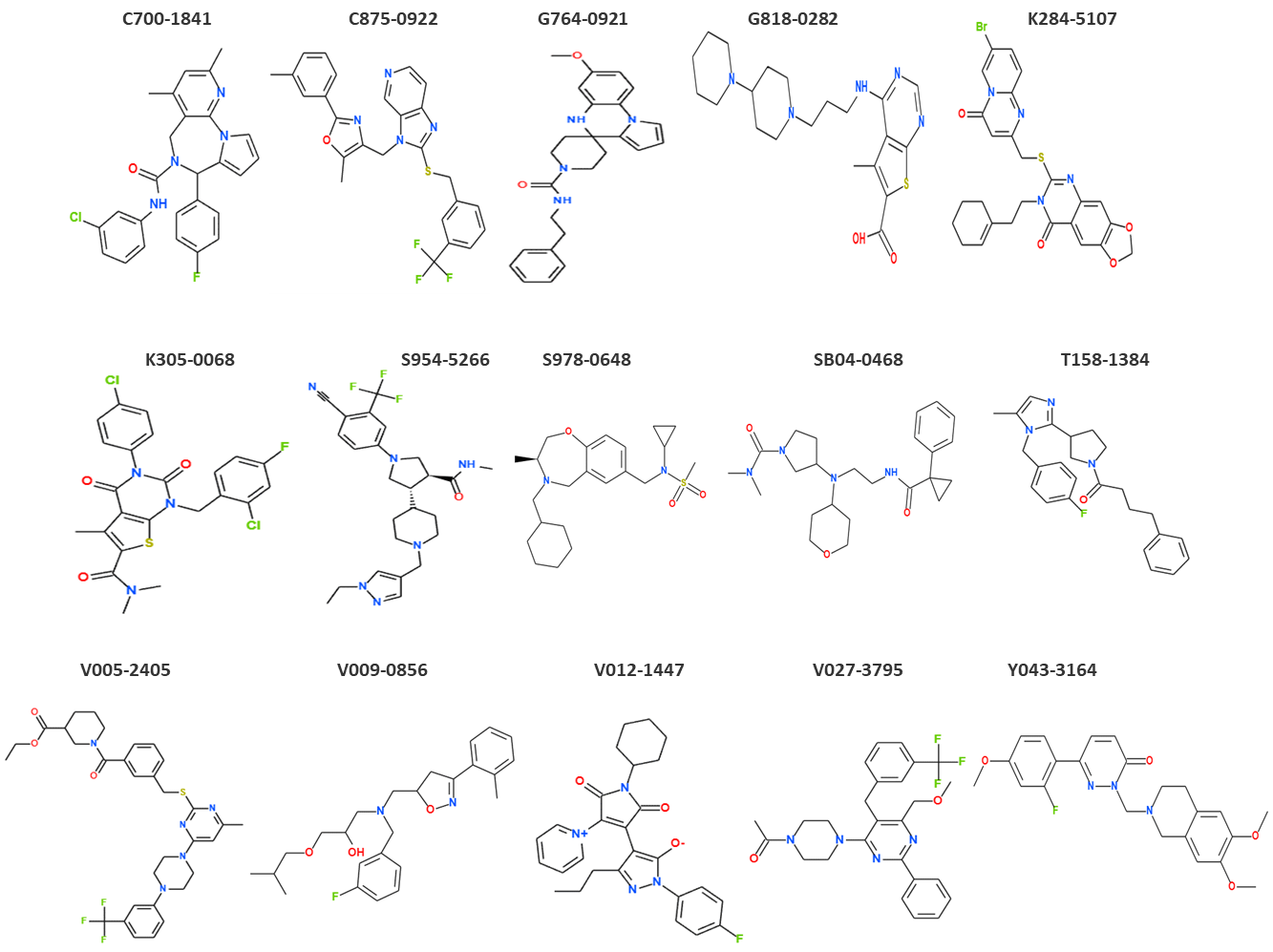
**

**Figure S5. Chemical structures of 15 selective candidates for GLP-1R.**

**
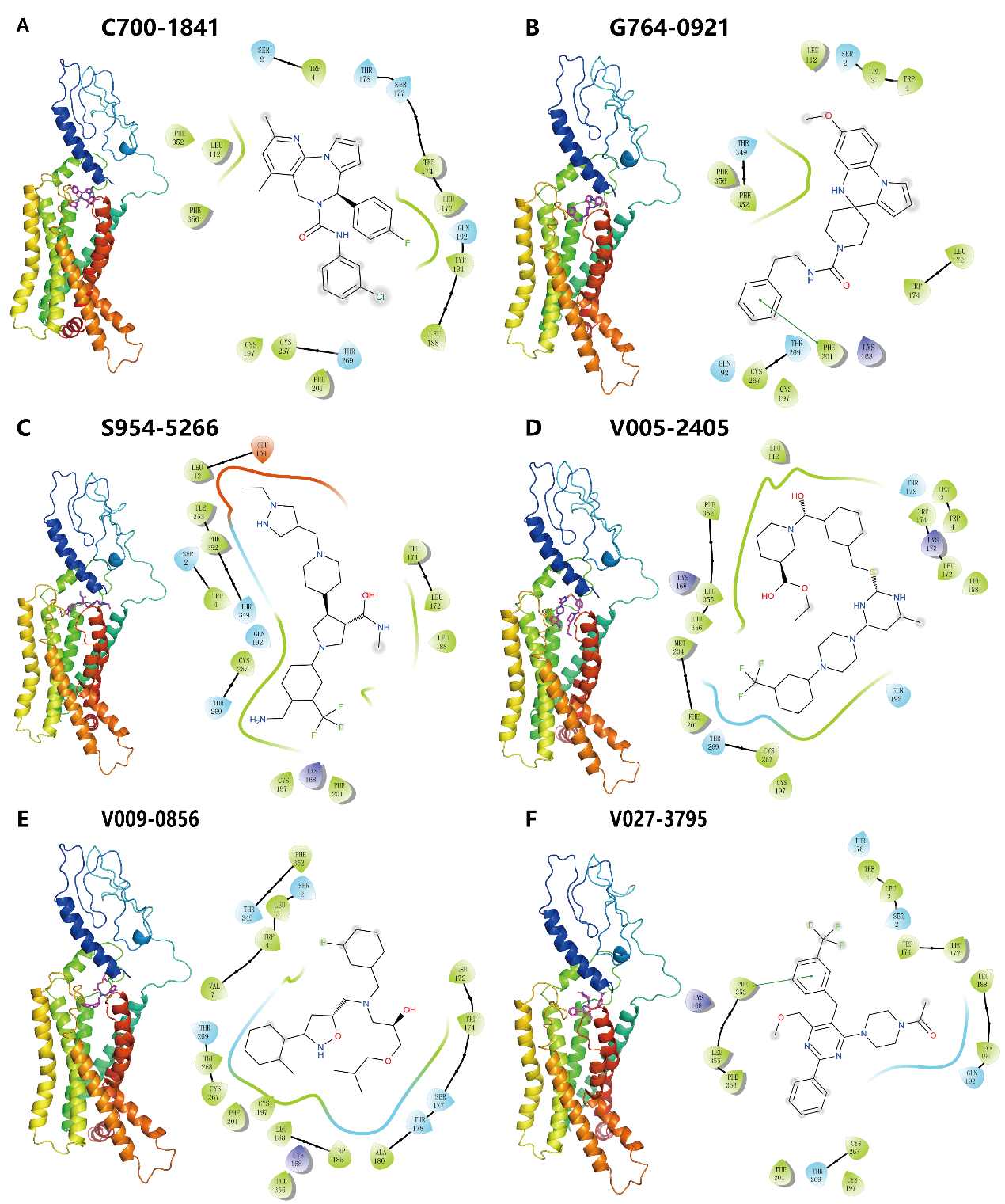
**

**Figure S6. the overall binding view and 2D plot of GLP_R1 with six active molecules from docking.** A, interactions between pocket residues and compound C700-1841. B. interactions between pocket residues and compound G764-0921. C. interactions between pocket residues and compound S954-5266. D. interactions between pocket residues and known active compound V005-2405. E. interactions between pocket residues and known active compound V009-0856. F. interactions between pocket residues and known active compound V027-3795.

**
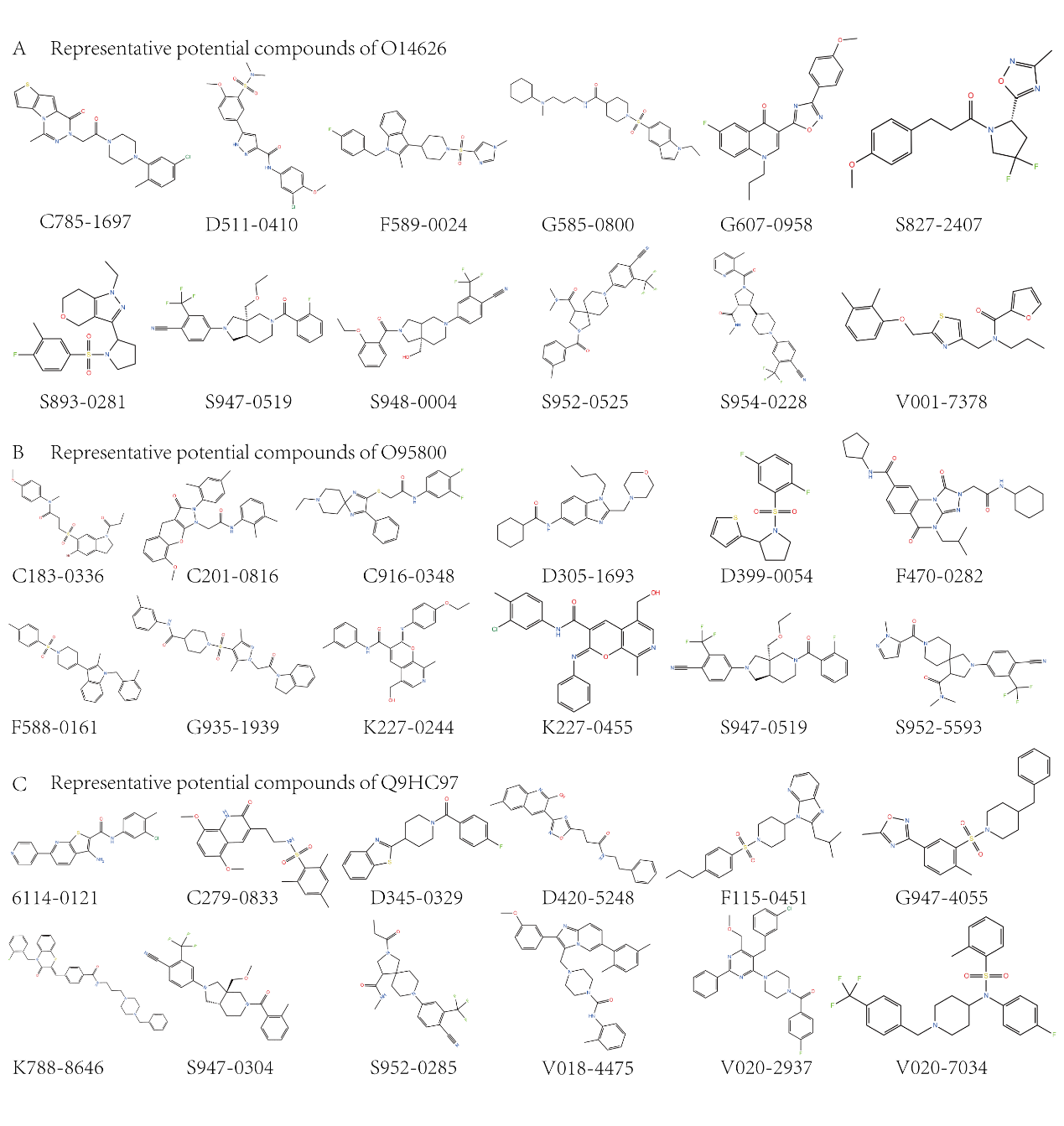
**

**Figure S7. The potential representative compounds of** **O14626,** **O95800, and** **Q9HC97, respectively.** A. Many of the representative compound candidates of O14626 show a linear shape. B. The structure of the representative compound candidates of O958000 is relatively diversified. C. The representative compounds candidates of Q9HC97, several representative structures contain common chemical groups, such as sulfonyl.

**
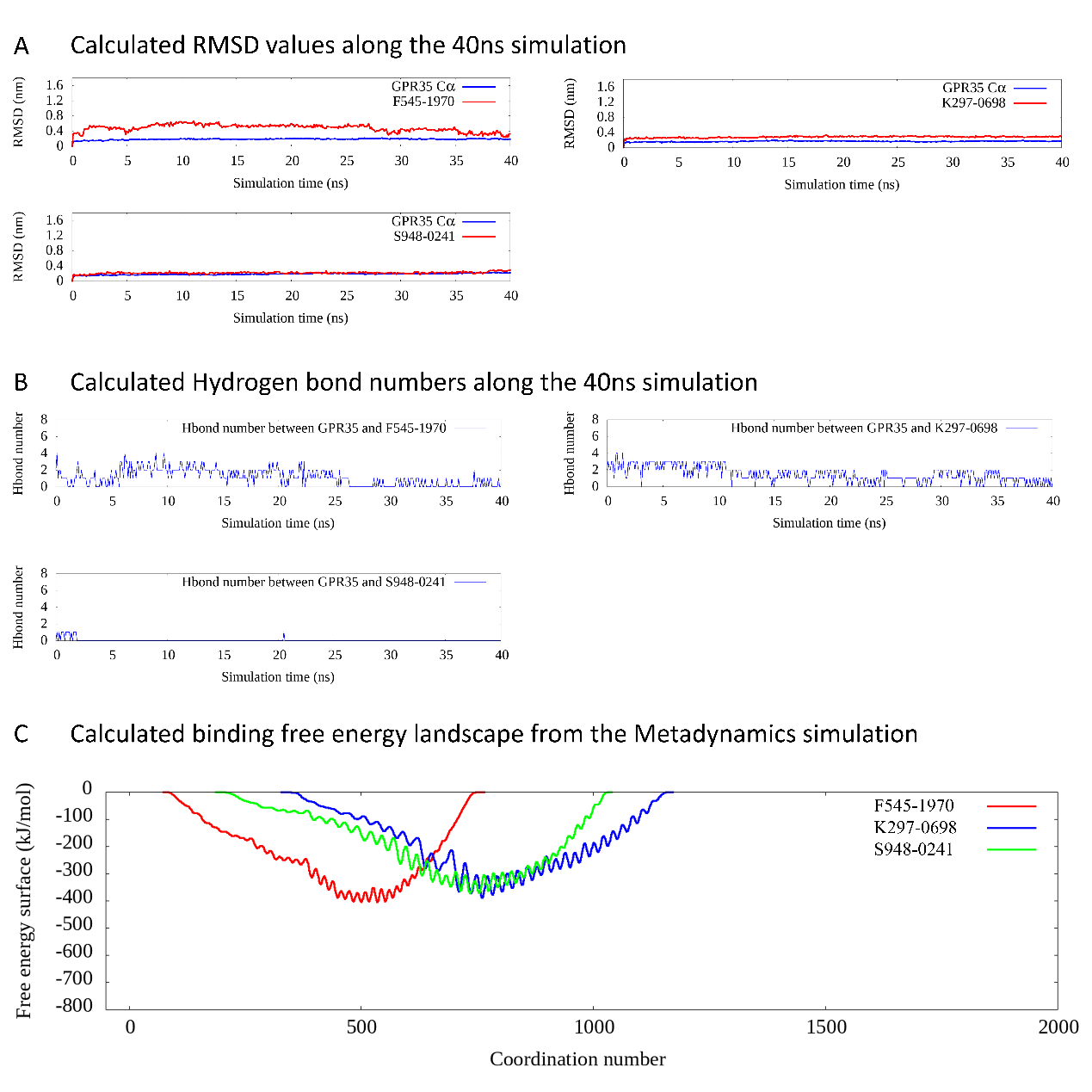
**

**Figure S8. The analysis result of MD and metadynamics simulation for GPR35 binding with the three compounds (F545-1970 K297-0698 and S948-0241).** A. The calculated RMSD value along the 40ns simulation time; B. The calculated hydrogen bond number along the 40ns simulation time; C. The calculated binding free energy landscape by metadynamics.


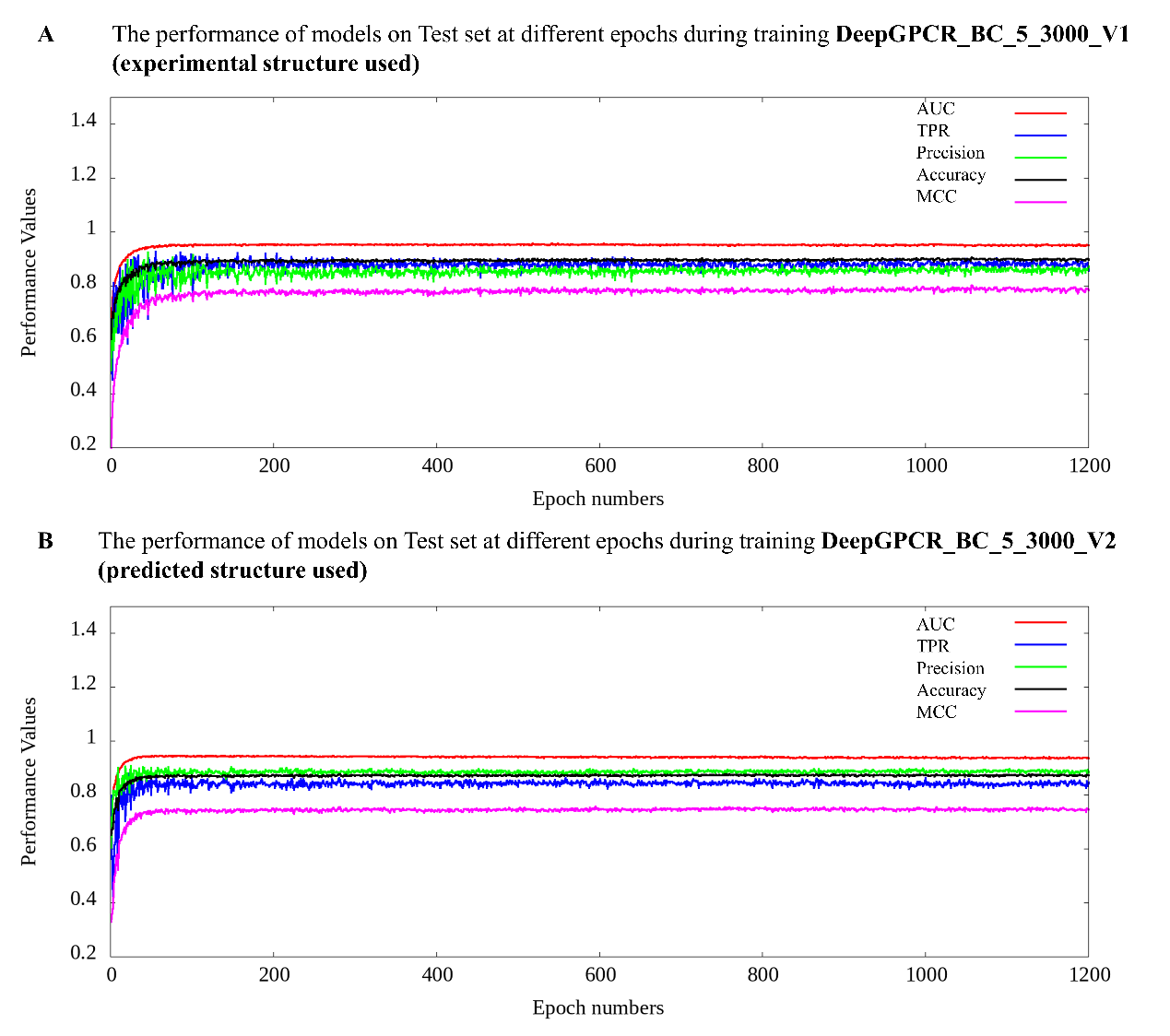


**Figure S9. Models developed with a 5 nM and 3000 nM cutoff for a positive/negative data split.** A. The performance of the models on the test set across various epochs during the training of DeepGPCR_BC_5_3000_V1 (using experimental structures); B. The performance of the models on the test set across various epochs during the training of DeepGPCR_BC_5_3000_V2 (using predicted structures).

**Supplementary Tables:**

**Table S1.** **The model performance on the training and test set1 at different training epochs, measured by AUC, TPR, precision, accuracy, and MCC.**

| **Dataset** | **epoch** | **AUC** | **TPR** | **Precision** | **Accuracy** | **MCC** |
| --- | --- | --- | --- | --- | --- | --- |
| Training set | 100 | 1.00 | 0.97 | 0.95 | 0.97 | 0.93 |
|  | 200 | 1.00 | 0.97 | 0.96 | 0.97 | 0.94 |
|  | 300 | 1.00 | 0.97 | 0.97 | 0.98 | 0.95 |
|  | 400 | 1.00 | 0.98 | 0.97 | 0.98 | 0.95 |
|  | 500 | 1.00 | 0.98 | 0.98 | 0.98 | 0.96 |
|  | 600 | 1.00 | 0.97 | 0.98 | 0.98 | 0.96 |
|  | 700 | 1.00 | 0.97 | 0.98 | 0.98 | 0.96 |
|  | 800 | 1.00 | 0.97 | 0.98 | 0.98 | 0.96 |
|  | 900 | 1.00 | 0.97 | 0.97 | 0.98 | 0.95 |
|  | 1000 | 1.00 | 0.97 | 0.99 | 0.98 | 0.96 |
|  | 1100 | 1.00 | 0.97 | 0.99 | 0.98 | 0.96 |
|  | 1200 | 1.00 | 0.97 | 0.98 | 0.98 | 0.96 |
|  | 1300 | 1.00 | 0.97 | 0.98 | 0.98 | 0.96 |
|  | 1400 | 1.00 | 0.97 | 0.98 | 0.98 | 0.96 |
|  | 1500 | 1.00 | 0.97 | 0.99 | 0.98 | 0.96 |
|  | 1600 | 1.00 | 0.97 | 0.99 | 0.98 | 0.96 |
|  | 1700 | 1.00 | 0.97 | 0.99 | 0.98 | 0.96 |
|  | 1800 | 1.00 | 0.97 | 0.99 | 0.98 | 0.96 |
|  | 1900 | 1.00 | 0.97 | 0.99 | 0.98 | 0.96 |
|  | 2000 | 1.00 | 0.98 | 0.98 | 0.98 | 0.96 |
| Test set1 | 100 | 0.96 | 0.92 | 0.87 | 0.91 | 0.82 |
|  | 200 | 0.97 | 0.90 | 0.87 | 0.91 | 0.81 |
|  | 300 | 0.97 | 0.90 | 0.89 | 0.92 | 0.82 |
|  | 400 | 0.97 | 0.92 | 0.86 | 0.91 | 0.82 |
|  | 500 | 0.97 | 0.90 | 0.89 | 0.92 | 0.83 |
|  | 600 | 0.97 | 0.90 | 0.88 | 0.91 | 0.82 |
|  | 700 | 0.97 | 0.90 | 0.89 | 0.92 | 0.83 |
|  | 800 | 0.97 | 0.88 | 0.89 | 0.91 | 0.82 |
|  | 900 | 0.97 | 0.91 | 0.87 | 0.92 | 0.82 |
|  | 1000 | 0.97 | 0.90 | 0.90 | 0.92 | 0.83 |
|  | 1100 | 0.97 | 0.89 | 0.91 | 0.92 | 0.84 |
|  | 1200 | 0.97 | 0.89 | 0.89 | 0.92 | 0.82 |
|  | 1300 | 0.96 | 0.90 | 0.88 | 0.91 | 0.82 |
|  | 1400 | 0.96 | 0.90 | 0.89 | 0.92 | 0.82 |
|  | 1500 | 0.97 | 0.90 | 0.90 | 0.92 | 0.84 |
|  | 1600 | 0.97 | 0.90 | 0.90 | 0.92 | 0.84 |
|  | 1700 | 0.96 | 0.89 | 0.90 | 0.92 | 0.83 |
|  | 1800 | 0.96 | 0.89 | 0.90 | 0.92 | 0.83 |
|  | 1900 | 0.96 | 0.90 | 0.90 | 0.92 | 0.83 |
|  | 2000 | 0.97 | 0.90 | 0.90 | 0.92 | 0.84 |

**Table S2. The performance on the test set2 using model at 2000th training epoch, measured by AUC, TPR, precision, accuracy, and MCC.** Notably, this set only contain protein P29274 related interaction.

| **AUC** | **TPR** | **Precision** | **Accuracy** | **MCC** | **pos_num** | **neg_num** |
| --- | --- | --- | --- | --- | --- | --- |
| 0.72 | 0.46 | 0.46 | 0.70 | 0.16 | 489 | 1179 |

**Table S3. The Autodock vina performance on an extra dataset with modeled GPCR protein and predicted pocket.** We used -6 Kcal/mol as the cutoff, those scores > -6 Kcal/mol was assigned a value of 0 (indicating non-bind), and those scores ≤ -6 Kcal/mol was assigned a value of 1 (indicating able to bind).

| **Name** | **AUC** | **TPR** | **precision** | **accuracy** | **MCC** | **F1 score** | **pos_num** | **neg_num** |
| --- | --- | --- | --- | --- | --- | --- | --- | --- |
| Q9Y5N1 | 0.63 | 0.81 | 0.90 | 0.76 | 0.21 | 0.86 | 1308 | 201 |
| P19327 | 0.59 | 0.65 | 0.75 | 0.61 | 0.18 | 0.70 | 948 | 451 |
| Q99705 | 0.58 | 0.96 | 0.53 | 0.57 | 0.25 | 0.68 | 450 | 483 |
| P41144 | 0.57 | 0.81 | 0.66 | 0.63 | 0.17 | 0.73 | 552 | 352 |
| P29275 | 0.57 | 1.00 | 0.18 | 0.27 | 0.16 | 0.30 | 147 | 794 |
| P20309 | 0.57 | 0.72 | 0.63 | 0.59 | 0.14 | 0.67 | 664 | 478 |
| P25099 | 0.54 | 0.98 | 0.21 | 0.27 | 0.12 | 0.35 | 199 | 813 |
| P21554 | 0.54 | 1.00 | 0.33 | 0.37 | 0.17 | 0.50 | 571 | 1238 |
| P0DMS8 | 0.54 | 0.98 | 0.43 | 0.46 | 0.16 | 0.60 | 566 | 805 |
| P50406 | 0.52 | 0.91 | 0.74 | 0.70 | 0.05 | 0.82 | 913 | 339 |
| P33535 | 0.51 | 0.96 | 0.68 | 0.67 | 0.04 | 0.79 | 913 | 443 |
| O43613 | 0.50 | 1.00 | 0.24 | 0.24 | 0.04 | 0.38 | 226 | 734 |
| P61169 | 0.48 | 0.74 | 0.39 | 0.43 | -0.04 | 0.51 | 529 | 805 |
| P35372 | 0.48 | 0.90 | 0.48 | 0.47 | -0.08 | 0.63 | 1054 | 1077 |
| P33533 | 0.46 | 0.02 | 0.24 | 0.37 | -0.19 | 0.04 | 609 | 399 |
| P32245 | 0.26 | 0.01 | 0.01 | 0.30 | -0.52 | 0.01 | 404 | 575 |
| ALL | 0.49 | 0.78 | 0.50 | 0.49 | -0.02 | 0.61 | 10053 | 9987 |

**Table S4. The Autodock vina performance on an extra dataset with modeled GPCR protein and predicted pocket (-5 Kcal/mol as the cutoff)**. Those scores > -5 Kcal/mol was assigned a value of 0 (indicating non-bind), and those scores ≤ -5 Kcal/mol was assigned a value of 1 (indicating able to bind).

| **Name** | **AUC** | **TPR** | **precision** | **accuracy** | **MCC** | **F1 score** | **pos_num** | **neg_num** |
| --- | --- | --- | --- | --- | --- | --- | --- | --- |
| P20309 | 0.53 | 0.75 | 0.60 | 0.57 | 0.07 | 0.67 | 664 | 478 |
| Q9Y5N1 | 0.53 | 0.96 | 0.87 | 0.85 | 0.10 | 0.92 | 1308 | 201 |
| P50406 | 0.53 | 1.00 | 0.74 | 0.74 | 0.18 | 0.85 | 913 | 339 |
| P19327 | 0.52 | 0.92 | 0.69 | 0.66 | 0.05 | 0.79 | 948 | 451 |
| P33535 | 0.51 | 0.97 | 0.68 | 0.67 | 0.07 | 0.80 | 913 | 443 |
| P29275 | 0.51 | 1.00 | 0.16 | 0.18 | 0.07 | 0.28 | 147 | 794 |
| P41144 | 0.51 | 0.91 | 0.62 | 0.60 | 0.04 | 0.73 | 552 | 352 |
| P0DMS8 | 0.51 | 0.98 | 0.42 | 0.43 | 0.07 | 0.59 | 566 | 805 |
| Q99705 | 0.51 | 0.98 | 0.49 | 0.49 | 0.05 | 0.65 | 450 | 483 |
| P25099 | 0.50 | 0.99 | 0.20 | 0.21 | 0.03 | 0.33 | 199 | 813 |
| P21554 | 0.50 | 1.00 | 0.32 | 0.32 | 0.05 | 0.48 | 571 | 1238 |
| O43613 | 0.50 | 1.00 | 0.24 | 0.24 | 0.04 | 0.38 | 226 | 734 |
| P35372 | 0.48 | 0.92 | 0.49 | 0.48 | -0.07 | 0.64 | 1054 | 1077 |
| P61169 | 0.48 | 0.91 | 0.39 | 0.39 | -0.07 | 0.54 | 529 | 805 |
| P33533 | 0.42 | 0.12 | 0.39 | 0.36 | -0.20 | 0.18 | 609 | 399 |
| P32245 | 0.20 | 0.02 | 0.03 | 0.23 | -0.61 | 0.03 | 404 | 575 |
| ALL | 0.48 | 0.86 | 0.49 | 0.48 | -0.07 | 0.62 | 10053 | 9987 |

**Table S5. The Autodock vina performance on an extra dataset with modeled GPCR protein and predicted pocket (-7 Kcal/mol as the cutoff)**. Those scores > -7 Kcal/mol was assigned a value of 0 (indicating non-bind), and those scores ≤ -7 Kcal/mol was assigned a value of 1 (indicating able to bind).

| **Name** | **AUC** | **TPR** | **precision** | **accuracy** | **MCC** | **F1 score** | **pos_num** | **neg_num** |
| --- | --- | --- | --- | --- | --- | --- | --- | --- |
| Q99705 | 0.67 | 0.67 | 0.65 | 0.67 | 0.33 | 0.66 | 450 | 483 |
| P29275 | 0.66 | 0.93 | 0.22 | 0.47 | 0.24 | 0.36 | 147 | 794 |
| P21554 | 0.62 | 0.95 | 0.38 | 0.49 | 0.27 | 0.54 | 571 | 1238 |
| P25099 | 0.60 | 0.94 | 0.24 | 0.39 | 0.19 | 0.38 | 199 | 813 |
| P20309 | 0.59 | 0.47 | 0.68 | 0.57 | 0.17 | 0.56 | 664 | 478 |
| P41144 | 0.57 | 0.35 | 0.72 | 0.52 | 0.15 | 0.47 | 552 | 352 |
| Q9Y5N1 | 0.56 | 0.32 | 0.92 | 0.38 | 0.10 | 0.47 | 1308 | 201 |
| P61169 | 0.55 | 0.43 | 0.46 | 0.57 | 0.10 | 0.45 | 529 | 805 |
| P50406 | 0.54 | 0.60 | 0.76 | 0.57 | 0.07 | 0.67 | 913 | 339 |
| P0DMS8 | 0.54 | 0.89 | 0.43 | 0.48 | 0.10 | 0.58 | 566 | 805 |
| O43613 | 0.52 | 0.99 | 0.24 | 0.27 | 0.08 | 0.39 | 226 | 734 |
| P33535 | 0.52 | 0.94 | 0.68 | 0.66 | 0.06 | 0.79 | 913 | 443 |
| P19327 | 0.50 | 0.07 | 0.66 | 0.35 | -0.01 | 0.13 | 948 | 451 |
| P33533 | 0.48 | 0.00 | 0.00 | 0.38 | -0.16 | 0.00 | 609 | 399 |
| P35372 | 0.48 | 0.87 | 0.48 | 0.47 | -0.07 | 0.62 | 1054 | 1077 |
| P32245 | 0.36 | 0.01 | 0.02 | 0.42 | -0.37 | 0.01 | 404 | 575 |
| ALL | 0.48 | 0.54 | 0.48 | 0.48 | -0.04 | 0.51 | 10053 | 9987 |

**Table S6.** Performance of DeepGPCR_RG for Training and test set at different epochs.

| **Dataset** | **epoch** | **rmse** | **mse** | **pearson** | **spearman** |
| --- | --- | --- | --- | --- | --- |
| Training | 200 | 0.72 | 0.51 | 0.84 | 0.83 |
|  | 400 | 0.68 | 0.46 | 0.86 | 0.85 |
|  | 600 | 0.66 | 0.44 | 0.87 | 0.86 |
|  | 800 | 0.66 | 0.44 | 0.87 | 0.86 |
|  | 1000 | 0.66 | 0.43 | 0.87 | 0.86 |
|  | 1200 | 0.65 | 0.42 | 0.87 | 0.86 |
|  | 1400 | 0.66 | 0.43 | 0.87 | 0.86 |
|  | 1600 | 0.65 | 0.42 | 0.87 | 0.86 |
|  | 1800 | 0.65 | 0.42 | 0.87 | 0.86 |
|  | 2000 | 0.64 | 0.41 | 0.87 | 0.86 |
| test | 200 | 0.98 | 0.96 | 0.68 | 0.67 |
|  | 400 | 1.00 | 1.01 | 0.67 | 0.65 |
|  | 600 | 1.01 | 1.03 | 0.66 | 0.64 |
|  | 800 | 1.04 | 1.09 | 0.66 | 0.64 |
|  | 1000 | 1.02 | 1.04 | 0.65 | 0.63 |
|  | 1200 | 1.04 | 1.09 | 0.65 | 0.63 |
|  | 1400 | 1.03 | 1.06 | 0.64 | 0.63 |
|  | 1600 | 1.04 | 1.08 | 0.64 | 0.63 |
|  | 1800 | 1.05 | 1.11 | 0.64 | 0.63 |
|  | 2000 | 1.05 | 1.11 | 0.64 | 0.63 |

**Table S7.** The Schrödinger docking performance on regression model’s extra test datasets.

| **Name** | **rmse** | **mse** | **pearson** | **spearman** | **total_num** |
| --- | --- | --- | --- | --- | --- |
| Q9Y5N1 | 4.93 | 24.31 | 0.16 | 0.17 | 3693 |
| P61169 | 3.55 | 12.59 | 0.10 | 0.08 | 4460 |
| P29275 | 2.43 | 5.91 | 0.09 | 0.17 | 1804 |
| P19327 | 3.85 | 14.83 | 0.06 | 0.09 | 4632 |
| P33535 | 3.92 | 15.39 | 0.02 | 0.02 | 2962 |
| P25099 | 1.83 | 3.33 | 0.01 | -0.01 | 2932 |
| P21554 | 3.73 | 13.94 | 0.01 | -0.02 | 3530 |
| P0DMS8 | 2.15 | 4.60 | 0.00 | -0.02 | 3797 |
| P20309 | 4.57 | 20.89 | -0.01 | 0.01 | 2006 |
| Q99705 | 4.34 | 18.86 | -0.02 | 0.05 | 3610 |
| P50406 | 3.78 | 14.30 | -0.02 | -0.01 | 3297 |
| O43613 | 5.73 | 32.86 | -0.06 | -0.06 | 3811 |
| P41144 | 5.10 | 26.00 | -0.08 | -0.02 | 2173 |
| P35372 | 3.62 | 13.10 | -0.09 | -0.06 | 4236 |
| P33533 | 7.62 | 58.10 | -0.13 | -0.12 | 1969 |
| P32245 | 7.01 | 49.17 | -0.30 | -0.26 | 2613 |
| Average | 4.37 | 19.11 | -0.04 | -0.04 | 51525 |

**Table S8.** The vina docking performance on regression model’s extra test datasets.

| **Name** | **rmse** | **mse** | **pearson** | **spearman** | **total_num** |
| --- | --- | --- | --- | --- | --- |
| O43613 | 2.02 | 4.09 | 0.02 | 0.11 | 3811 |
| P19327 | 3.22 | 10.39 | 0.05 | 0.06 | 4632 |
| P21554 | 1.81 | 3.28 | 0.25 | 0.26 | 3530 |
| P29275 | 1.71 | 2.94 | 0.31 | 0.36 | 1804 |
| P33533 | 10.75 | 115.46 | 0.08 | -0.11 | 1969 |
| P41144 | 3.30 | 10.88 | 0.05 | 0.04 | 2173 |
| Q99705 | 2.31 | 5.32 | 0.18 | 0.18 | 3610 |
| P33535 | 3.73 | 13.91 | -0.01 | 0.05 | 2962 |
| P50406 | 2.56 | 6.57 | 0.02 | 0.02 | 3297 |
| Q9Y5N1 | 3.40 | 11.59 | 0.19 | 0.21 | 3693 |
| P0DMS8 | 2.02 | 4.10 | -0.04 | -0.08 | 3797 |
| P20309 | 4.13 | 17.02 | 0.03 | 0.10 | 2006 |
| P25099 | 1.62 | 2.64 | 0.13 | 0.13 | 2932 |
| P32245 | 15.00 | 225.02 | -0.29 | -0.42 | 2613 |
| P35372 | 4.13 | 17.09 | -0.05 | -0.03 | 4236 |
| P61169 | 2.40 | 5.77 | 0.03 | 0.08 | 4460 |
| Average | 4.83 | 23.29 | -0.06 | -0.07 | 51525 |

**Table S9.** The compound list of GPR35 by using DeepGPCR_RG and Schrödinger (DeepGPCR_RG≥10, Schrödinger score≤-6.35 Kcal/mol).

| **Chemdiv id** | **DeepGPCR_RG** | **Schrödinger**  **(Kcal/mol)** |
| --- | --- | --- |
| V010-1264 | 10.03 | -7.02 |
| 6028-3969 | 10.30 | -6.77 |
| E146-0380 | 10.45 | -6.47 |
| E014-0043 | 12.16 | -6.40 |
| D103-0816 | 10.10 | -6.39 |
| L311-0042 | 10.18 | -6.35 |

**Table S10.** The compound list of GLP_1R by using DeepGPCR_RG and Schrödinger (DeepGPCR_RG≥10.5, Schrödinger score≤-8.7 Kcal/mol).

| **Chemdiv id** | **DeepGPCR_RG** | **Schrödinger**  **(Kcal/mol)** |
| --- | --- | --- |
| G764-0921 | 10.80 | -8.71 |
| K284-5107 | 10.71 | -8.90 |
| K305-0068 | 10.61 | -8.79 |

**Table S11.** DFCNN performance on an extra dataset with modeled GPCR protein and predicted pocket.

| **Name** | **AUC** | **TPR** | **precision** | **accuracy** | **MCC** | **F1 score** | **pos_num** | **neg_num** |
| --- | --- | --- | --- | --- | --- | --- | --- | --- |
| P29275 | 0.75 | 0.56 | 0.47 | 0.83 | 0.42 | 0.52 | 147 | 794 |
| P35372 | 0.66 | 0.42 | 0.65 | 0.61 | 0.22 | 0.51 | 1054 | 1105 |
| P21554 | 0.63 | 0.02 | 0.09 | 0.63 | -0.13 | 0.03 | 571 | 1242 |
| P25099 | 0.60 | 0.25 | 0.20 | 0.66 | 0.01 | 0.22 | 199 | 833 |
| P0DMS8 | 0.57 | 0.14 | 0.32 | 0.53 | -0.08 | 0.19 | 566 | 813 |
| P33533 | 0.57 | 0.63 | 0.63 | 0.56 | 0.09 | 0.63 | 609 | 409 |
| P61169 | 0.55 | 0.61 | 0.43 | 0.53 | 0.09 | 0.51 | 529 | 817 |
| Q9Y5N1 | 0.51 | 0.29 | 0.82 | 0.34 | -0.07 | 0.43 | 1308 | 219 |
| P32245 | 0.50 | 0.38 | 0.43 | 0.53 | 0.02 | 0.40 | 404 | 575 |
| P33535 | 0.49 | 0.33 | 0.61 | 0.41 | -0.10 | 0.43 | 913 | 449 |
| P50406 | 0.48 | 0.25 | 0.73 | 0.38 | 0.00 | 0.37 | 913 | 339 |
| Q99705 | 0.43 | 0.76 | 0.46 | 0.46 | -0.07 | 0.58 | 450 | 485 |
| P19327 | 0.37 | 0.19 | 0.50 | 0.32 | -0.23 | 0.28 | 948 | 453 |
| P41144 | 0.33 | 0.06 | 0.49 | 0.39 | -0.07 | 0.11 | 552 | 362 |
| O43613 | 0.23 | 0.10 | 0.07 | 0.45 | -0.30 | 0.08 | 226 | 734 |
| P20309 | 0.14 | 0.10 | 0.19 | 0.24 | -0.52 | 0.13 | 664 | 492 |
| ALL | 0.55 | 0.31 | 0.49 | 0.49 | -0.01 | 0.38 | 10053 | 10121 |

**Table S12.** DeepBindGCN_BC performance on an extra dataset with modeled GPCR protein and predicted pocket.

| **Name** | **AUC** | **TPR** | **precision** | **accuracy** | **MCC** | **F1 score** | **pos_num** | **neg_num** |
| --- | --- | --- | --- | --- | --- | --- | --- | --- |
| P29275 | 0.75 | 0.56 | 0.47 | 0.83 | 0.42 | 0.52 | 147 | 794 |
| P35372 | 0.66 | 0.42 | 0.65 | 0.61 | 0.22 | 0.51 | 1054 | 1105 |
| P21554 | 0.63 | 0.02 | 0.09 | 0.63 | -0.13 | 0.03 | 571 | 1242 |
| P25099 | 0.60 | 0.25 | 0.20 | 0.66 | 0.01 | 0.22 | 199 | 833 |
| P0DMS8 | 0.57 | 0.14 | 0.32 | 0.53 | -0.08 | 0.19 | 566 | 813 |
| P33533 | 0.57 | 0.63 | 0.63 | 0.56 | 0.09 | 0.63 | 609 | 409 |
| P61169 | 0.55 | 0.61 | 0.43 | 0.53 | 0.09 | 0.51 | 529 | 817 |
| Q9Y5N1 | 0.51 | 0.29 | 0.82 | 0.34 | -0.07 | 0.43 | 1308 | 219 |
| P32245 | 0.50 | 0.38 | 0.43 | 0.53 | 0.02 | 0.40 | 404 | 575 |
| P33535 | 0.49 | 0.33 | 0.61 | 0.41 | -0.10 | 0.43 | 913 | 449 |
| P50406 | 0.48 | 0.25 | 0.73 | 0.38 | 0.00 | 0.37 | 913 | 339 |
| Q99705 | 0.43 | 0.76 | 0.46 | 0.46 | -0.07 | 0.58 | 450 | 485 |
| P19327 | 0.37 | 0.19 | 0.50 | 0.32 | -0.23 | 0.28 | 948 | 453 |
| P41144 | 0.33 | 0.06 | 0.49 | 0.39 | -0.07 | 0.11 | 552 | 362 |
| O43613 | 0.23 | 0.10 | 0.07 | 0.45 | -0.30 | 0.08 | 226 | 734 |
| P20309 | 0.14 | 0.10 | 0.19 | 0.24 | -0.52 | 0.13 | 664 | 492 |
| ALL | 0.55 | 0.31 | 0.49 | 0.49 | -0.01 | 0.38 | 10053 | 10121 |

**Table S13. DeepBindGCN_RG performance on the 16-protein related extra dataset.**

| **Name** | **rmse** | **mse** | **pearson** | **spearman** | **total_num** |
| --- | --- | --- | --- | --- | --- |
| P29275 | 1.27 | 1.62 | 0.31 | 0.29 | 1804 |
| Q9Y5N1 | 1.30 | 1.69 | 0.10 | 0.12 | 3693 |
| P50406 | 1.45 | 2.11 | 0.11 | 0.10 | 3297 |
| P25099 | 1.67 | 2.80 | 0.08 | 0.09 | 2932 |
| P35372 | 1.66 | 2.75 | 0.27 | 0.27 | 4236 |
| O43613 | 1.16 | 1.35 | 0.20 | 0.20 | 3811 |
| P0DMS8 | 1.45 | 2.09 | 0.21 | 0.20 | 3797 |
| P21554 | 1.15 | 1.33 | 0.31 | 0.29 | 3530 |
| P33533 | 1.54 | 2.37 | 0.08 | 0.07 | 1969 |
| P41144 | 1.30 | 1.70 | 0.28 | 0.28 | 2173 |
| P32245 | 1.52 | 2.31 | 0.22 | 0.20 | 2613 |
| P20309 | 1.68 | 2.83 | 0.28 | 0.29 | 2006 |
| Q99705 | 1.25 | 1.56 | 0.22 | 0.22 | 3610 |
| P33535 | 1.54 | 2.37 | 0.20 | 0.21 | 2962 |
| P61169 | 1.32 | 1.75 | -0.01 | -0.02 | 4460 |
| P19327 | 1.63 | 2.65 | 0.07 | 0.06 | 4632 |
| Average | 1.44 | 2.07 | 0.20 | 0.19 | 51525 |

**Table S14. The representative screening result for target O14626, O95800, Q9HC97 by DeepGPCR and Schrödinger.**

| **Target** | **Name** | **DeepGPCR score** | **Schrödinger score (Kcal/mol)** |
| --- | --- | --- | --- |
| O14626 | 8015-6811 | 1 | -8.02 |
|  | T842-2224 | 1 | -6.97 |
|  | SC41-0196 | 1 | -6.9 |
|  | V007-8842 | 1 | -6.83 |
|  | S828-3346 | 1 | -6.6 |
|  | D399-0391 | 1 | -6.42 |
|  | SA92-0434 | 1 | -6.33 |
|  | J004-1117 | 1 | -6.19 |
|  | SC41-0276 | 1 | -6.13 |
|  | P094-1462 | 1 | -6.04 |
|  | SA70-0617 | 1 | -6 |
|  | F892-0669 | 1 | -6 |
|  | D399-0532 | 1 | -5.94 |
|  | E565-0464 | 1 | -5.92 |
|  | S827-4083 | 1 | -5.89 |
|  | M621-0356 | 1 | -5.88 |
| O95800 | 8019-4552 | 1 | -5.69 |
|  | 8020-4415 | 1 | -4.49 |
|  | 0708-0003 | 1 | -4.33 |
|  | 8011-6477 | 1 | -3.13 |
|  | Y031-6963 | 1 | -2.77 |
|  | 8020-6929 | 1 | -2.45 |
|  | 0896-4678 | 1 | -0.53 |
|  | 3137-0411 | 1 | -0.16 |
| Q9HC97 | K788-9238 | 1 | -8.07 |
|  | V030-8466 | 1 | -7.58 |
|  | K784-5585 | 1 | -7.47 |
|  | C529-0941 | 1 | -7.44 |
|  | S947-5093 | 1 | -7.41 |
|  | D233-0341 | 1 | -7.2 |
|  | S953-0095 | 1 | -7.16 |
|  | V004-3123 | 1 | -7.12 |
|  | C530-1213 | 1 | -7.12 |
|  | L310-0069 | 1 | -6.99 |
|  | C530-1173 | 1 | -6.96 |
|  | M019-1847 | 1 | -6.8 |
|  | D351-0870 | 1 | -6.79 |
|  | C530-1331 | 1 | -6.79 |
|  | S952-0152 | 1 | -6.77 |
|  | S957-0130 | 1 | -6.76 |
|  | M678-0235 | 1 | -6.75 |
|  | E535-0928 | 1 | -6.74 |
|  | T160-0582 | 1 | -6.74 |

**Table S15. The RMSD, TM-score between Alphafold2 predicted structure (Protein 1) and experimental PDB structure (Protein 2) for 62 selected GPCR.** Here, we only selected sequence identity (ID)>=0.83.

| **Protein 1**  **(F1-model_v4)** | **Protein 2**  **(From PDB)** | **Aligned length** | **RMSD** | **TM-score** | **ID** |
| --- | --- | --- | --- | --- | --- |
| AF-Q14416 | Q14416_4XAQ | 443 | 0.87 | 0.99 | 0.99 |
| AF-P41146 | P41146_4EA3 | 278 | 0.91 | 0.98 | 1.00 |
| AF-Q9Y271 | Q9Y271_6RZ4 | 290 | 1.23 | 0.72 | 0.99 |
| AF-P43220 | P43220_3C5T | 104 | 1.24 | 0.94 | 0.98 |
| AF-P21453 | P21453_3V2W | 295 | 1.27 | 0.65 | 0.92 |
| AF-P25103 | P25103_6E59 | 292 | 1.31 | 0.59 | 0.92 |
| AF-Q9UBS5 | Q9UBS5_4MQF | 406 | 1.34 | 0.97 | 1.00 |
| AF-P51686 | P51686_5LWE | 266 | 1.38 | 0.93 | 0.92 |
| AF-P41145 | P41145_6VI4 | 283 | 1.40 | 0.97 | 0.98 |
| AF-P47900 | P47900_4XNW | 297 | 1.50 | 0.83 | 0.98 |
| AF-P35408 | P35408_5YHL | 280 | 1.54 | 0.96 | 0.98 |
| AF-P51681 | P51681_4MBS | 295 | 1.57 | 0.82 | 0.95 |
| AF-P34972 | P34972_5ZTY | 301 | 1.60 | 0.65 | 0.94 |
| AF-Q92633 | Q92633_4Z34 | 307 | 1.62 | 0.77 | 0.93 |
| AF-Q9Y5Y4 | Q9Y5Y4_6D26 | 322 | 1.70 | 0.70 | 0.98 |
| AF-P31422 | P31422_2E4Y | 517 | 1.76 | 0.96 | 0.98 |
| AF-P30542 | P30542_5N2S | 284 | 1.78 | 0.72 | 0.95 |
| AF-P56726 | P56726_6O3C | 483 | 1.78 | 0.96 | 0.95 |
| AF-P29274 | P29274_2YDO | 295 | 1.79 | 0.90 | 0.98 |
| AF-P61073 | P61073_3OE9 | 270 | 1.81 | 0.62 | 0.94 |
| AF-P32300 | P32300_4EJ4 | 281 | 1.86 | 0.62 | 0.99 |
| AF-Q99835 | Q99835_4N4W | 357 | 1.87 | 0.76 | 0.97 |
| AF-Q92847 | Q92847_6KO5 | 293 | 1.88 | 0.70 | 0.97 |
| AF-P41594 | P41594_4OO9 | 244 | 1.88 | 0.58 | 0.94 |
| AF-P20789 | P20789_4XEE | 311 | 1.90 | 0.65 | 0.95 |
| AF-P25929 | P25929_5ZBQ | 285 | 1.92 | 0.59 | 0.93 |
| AF-P55085 | P55085_5NDD | 316 | 1.93 | 0.54 | 0.91 |
| AF-O43614 | O43614_4S0V | 319 | 1.96 | 0.64 | 0.88 |
| AF-P07550 | P07550_3D4S | 300 | 2.00 | 0.66 | 0.92 |
| AF-O00222 | O00222_6BT5 | 440 | 2.04 | 0.94 | 0.99 |
| AF-P28223 | P28223_6WHA | 244 | 2.05 | 0.91 | 0.96 |
| AF-P50052 | P50052_5UNF | 290 | 2.06 | 0.72 | 0.96 |
| AF-P32248 | P32248_6QZH | 276 | 2.07 | 0.36 | 0.93 |
| AF-P28222 | P28222_4IAQ | 309 | 2.17 | 0.80 | 0.87 |
| AF-P48039 | P48039_6ME2 | 297 | 2.17 | 0.59 | 0.87 |
| AF-P43116 | P43116_7CX2 | 265 | 2.19 | 0.91 | 0.96 |
| AF-P21730 | P21730_6C1Q | 299 | 2.19 | 0.76 | 0.95 |
| AF-P49286 | P49286_6ME6 | 308 | 2.19 | 0.66 | 0.89 |
| AF-P42866 | P42866_4DKL | 287 | 2.37 | 0.61 | 0.97 |
| AF-P51436 | P51436_6IQL | 275 | 2.37 | 0.75 | 0.86 |
| AF-P25090 | P25090_6LW5 | 302 | 2.38 | 0.67 | 0.97 |
| AF-P23385 | P23385_1ISS | 452 | 2.42 | 0.92 | 1.00 |
| AF-P30556 | P30556_4YAY | 285 | 2.45 | 0.67 | 0.93 |
| AF-P24530 | P24530_5XPR | 310 | 2.49 | 0.70 | 0.96 |
| AF-P35400 | P35400_2E4Z | 434 | 2.50 | 0.91 | 0.95 |
| AF-P32238 | P32238_7F8U | 314 | 2.52 | 0.67 | 0.88 |
| AF-Q9H244 | Q9H244_4NTJ | 272 | 2.65 | 0.67 | 0.89 |
| AF-P21917 | P21917_5WIV | 290 | 2.70 | 0.71 | 0.91 |
| AF-P08912 | P08912_6OL9 | 320 | 2.71 | 0.72 | 0.88 |
| AF-Q14832 | Q14832_3SM9 | 427 | 2.73 | 0.88 | 0.84 |
| AF-P30968 | P30968_7BR3 | 278 | 2.74 | 0.54 | 0.94 |
| AF-P41595 | P41595_5TUD | 322 | 2.79 | 0.78 | 0.87 |
| AF-P41143 | P41143_6PT3 | 301 | 2.84 | 0.69 | 0.93 |
| AF-P28335 | P28335_6BQG | 315 | 2.88 | 0.76 | 0.89 |
| AF-Q13255 | Q13255_3KS9 | 435 | 2.95 | 0.87 | 0.84 |
| AF-P08172 | P08172_3UON | 302 | 3.07 | 0.64 | 0.91 |
| AF-P08588 | P08588_7BTS | 307 | 3.21 | 0.62 | 0.90 |
| AF-P08483 | P08483_4U14 | 319 | 3.55 | 0.65 | 0.84 |
| AF-P14416 | P14416_6CM4 | 306 | 3.64 | 0.64 | 0.83 |
| AF-P35367 | P35367_3RZE | 311 | 3.70 | 0.65 | 0.85 |
| AF-Q14833 | Q14833_7E9H | 766 | 3.82 | 0.87 | 0.99 |
| AF-P47871 | P47871_5XEZ | 307 | 4.38 | 0.47 | 0.83 |
| Average | | 322.24 | 2.19 | 0.74 | 0.93 |

**Table S16. Performance of the trained DeepGPCR_BC_5_3000_V1 model on an independent test set of 16 proteins. For this evaluation, we utilized the model from epoch 1000.** The positive dataset was defined by affinities smaller than 5 nM, and the negative dataset was defined by affinities greater than 3000 nM. During training, all binding pockets were derived from known structures in the PDB database, as determined by known ligands (using experimental structures).

| **Name** | **AUC** | **TPR** | **precision** | **accuracy** | **MCC** | **F1 score** | **pos_num** | **neg_num** |
| --- | --- | --- | --- | --- | --- | --- | --- | --- |
| P35372 | 0.92 | 0.85 | 0.86 | 0.87 | 0.73 | 0.86 | 1129 | 1232 |
| P20309 | 0.91 | 0.85 | 0.91 | 0.87 | 0.74 | 0.88 | 706 | 527 |
| P61169 | 0.83 | 0.82 | 0.65 | 0.76 | 0.53 | 0.73 | 606 | 928 |
| O43613 | 0.79 | 0.69 | 0.48 | 0.75 | 0.41 | 0.56 | 261 | 865 |
| P19327 | 0.78 | 0.72 | 0.85 | 0.72 | 0.42 | 0.78 | 1052 | 502 |
| P33533 | 0.76 | 0.86 | 0.77 | 0.76 | 0.48 | 0.81 | 683 | 445 |
| P41144 | 0.75 | 0.81 | 0.72 | 0.71 | 0.38 | 0.76 | 595 | 429 |
| P25099 | 0.71 | 0.37 | 0.51 | 0.80 | 0.31 | 0.43 | 237 | 915 |
| P33535 | 0.65 | 0.72 | 0.72 | 0.64 | 0.20 | 0.72 | 971 | 517 |
| P32245 | 0.63 | 0.37 | 0.59 | 0.62 | 0.20 | 0.46 | 470 | 629 |
| Q99705 | 0.62 | 0.31 | 0.62 | 0.56 | 0.14 | 0.42 | 565 | 552 |
| Q9Y5N1 | 0.58 | 0.24 | 0.89 | 0.32 | 0.05 | 0.38 | 1416 | 233 |
| P29275 | 0.57 | 0.20 | 0.28 | 0.77 | 0.11 | 0.24 | 176 | 832 |
| P0DMS8 | 0.53 | 0.13 | 0.42 | 0.57 | 0.01 | 0.20 | 634 | 913 |
| P50406 | 0.50 | 0.15 | 0.78 | 0.35 | 0.05 | 0.26 | 999 | 378 |
| P21554 | 0.43 | 0.24 | 0.31 | 0.60 | 0.00 | 0.27 | 632 | 1441 |
| ALL | 0.70 | 0.54 | 0.71 | 0.66 | 0.34 | 0.61 | 11132 | 11338 |

**Table S17. Performance of the trained DeepGPCR_BC_5_3000_V2 model on an independent test set of 16 proteins.** For this assessment, we employed the epoch 1000 model. The positive data set was defined by affinities smaller than 5 nM, while the negative data set was defined by affinities greater than 3000 nM. Throughout training, all binding pockets were sourced from the AlphaFold2 protein database, with cofactors utilized to identify potential binding ligands. The predicted binding ligands closest to the N-terminal of the GPCR were chosen as pockets (using predicted structures).

| **Name** | **AUC** | **TPR** | **precision** | **accuracy** | **MCC** | **F1 score** | **pos_num** | **neg_num** |
| --- | --- | --- | --- | --- | --- | --- | --- | --- |
| P35372 | 0.92 | 0.86 | 0.84 | 0.86 | 0.72 | 0.85 | 1129 | 1232 |
| P20309 | 0.91 | 0.85 | 0.91 | 0.86 | 0.73 | 0.88 | 706 | 527 |
| P61169 | 0.84 | 0.77 | 0.70 | 0.78 | 0.55 | 0.74 | 606 | 928 |
| P25099 | 0.83 | 0.59 | 0.67 | 0.85 | 0.53 | 0.62 | 237 | 915 |
| P19327 | 0.82 | 0.71 | 0.88 | 0.73 | 0.47 | 0.78 | 1052 | 502 |
| P41144 | 0.82 | 0.86 | 0.75 | 0.76 | 0.49 | 0.80 | 595 | 429 |
| P32245 | 0.78 | 0.61 | 0.78 | 0.76 | 0.50 | 0.68 | 470 | 629 |
| P33535 | 0.77 | 0.77 | 0.79 | 0.72 | 0.39 | 0.78 | 971 | 517 |
| O43613 | 0.75 | 0.63 | 0.43 | 0.72 | 0.34 | 0.51 | 261 | 865 |
| Q99705 | 0.73 | 0.54 | 0.69 | 0.65 | 0.30 | 0.61 | 565 | 552 |
| Q9Y5N1 | 0.72 | 0.63 | 0.93 | 0.64 | 0.23 | 0.75 | 1416 | 233 |
| P33533 | 0.70 | 0.72 | 0.72 | 0.66 | 0.29 | 0.72 | 683 | 445 |
| P29275 | 0.70 | 0.24 | 0.27 | 0.75 | 0.10 | 0.25 | 176 | 832 |
| P0DMS8 | 0.67 | 0.32 | 0.66 | 0.65 | 0.25 | 0.43 | 634 | 913 |
| P21554 | 0.66 | 0.44 | 0.44 | 0.66 | 0.19 | 0.44 | 632 | 1441 |
| P50406 | 0.64 | 0.33 | 0.88 | 0.48 | 0.22 | 0.48 | 999 | 378 |
| ALL | 0.79 | 0.64 | 0.76 | 0.72 | 0.45 | 0.70 | 11132 | 11338 |
